# The use of public performance reporting by general practitioners: a study of perceptions and referral behaviours

**DOI:** 10.1186/s12875-018-0719-4

**Published:** 2018-02-12

**Authors:** Khic-Houy Prang, Rachel Canaway, Marie Bismark, David Dunt, Margaret Kelaher

**Affiliations:** Centre for Health Policy, Melbourne School of Population and Global Health, The University of Melbourne, Level 4, 207 Bouverie Street, Carlton, VIC 3010 Australia

**Keywords:** Public performance reporting, General practitioner, Primary care, Qualitative research

## Abstract

**Background:**

Public performance reporting (PPR) of hospital data aims to improve quality of care in hospitals and to inform consumer choice. In Australia, general practitioners (GPs) are gatekeepers to secondary care with patients requiring their referral for non-emergency access. Despite their intermediary role, GPs have been generally overlooked as potential users of PPR of hospital data, with the majority of the PPR research focussing on consumers, surgeons and hospitals.

**Methods:**

We examined the use of PPR of hospital data by GPs when referring patients to hospitals. Semi-structured interviews were conducted with 40 GPs, recruited via the Victorian Primary Care Practice-Based Research Network and GP teaching practices in Victoria, Australia. The interviews were recorded, transcribed and analysed thematically.

**Results:**

We found that the majority of GPs did not use PPR when referring patients to hospitals. Instead, they relied mostly on informal sources of information such as their own or patients’ previous experiences. Barriers that prevented GPs’ use of PPR in their decision making included: lack of awareness and accessibility; perceived lack of data credibility; restrictive geographical catchments for certain hospitals; limited choices of public hospitals in regional and rural areas; and no mandatory PPR for private hospitals.

**Conclusions:**

Our findings suggest that lack of PPR awareness prevented GPs from using it in their referral practice. As gatekeepers to secondary care, GPs are in a position to guide patients in their treatment decisions and referrals using available PPR data. We suggest that there needs to be greater involvement by GPs in the development of hospital performance and quality indicators in Australia if GPs are to make greater use of them. The indicators require further development before GPs perceive them as valid, credible, and of use for informing their referral practices.

**Electronic supplementary material:**

The online version of this article (10.1186/s12875-018-0719-4) contains supplementary material, which is available to authorized users.

## Background

Public release of performance information is widely discussed as a mechanism to improve transparency, accountability, choice, and quality of healthcare [1, 2]. Quality indicators and forms of public performance reporting (PPR) vary across different healthcare systems and countries. For example, PPR in the United States (US) includes publishing quality indicators such as mortality and complication rates in various forms - report cards, provider profiles and consumer reports [3]. In the United Kingdom (UK), ratings of individual specialists working in hospitals are publicly reported; and in many countries, data on patients’ experiences of hospital care is routinely available [2]. In Australia, mandatory PPR of public hospital data was introduced in 2011, with the establishment of the National Health Performance Authority (ceased in 2016 and activities transferred to the Australian Institute of Health and Welfare [AIHW] thereafter) and the launch of the MyHospitals website [4]. Indicators reported on the MyHospitals website include staphylococcus aureus infections, time patients spent in emergency department, cancer surgery waiting times and financial performance of public hospitals. Indicators yet to be publicly reported, due to their associated methodological issues, include measures of mortality, unplanned readmission rates, patient experiences and access to services by type of service compared to need. Reporting to MyHospitals is mandatory for Australian public hospitals but voluntary for private hospitals. There are also other state/territory government and institution-based sources of PPR – such as the Victorian Health Services Performance website [5].

Berwick and colleagues [6] hypothesised that PPR improves quality of care through two pathways: 1) the selection pathway and 2) the quality change pathway. In the selection pathway, consumers compare PPR data and select high quality healthcare providers and services. This leads to growth of high quality providers, and contraction or exit of low quality providers. The change pathway encourages healthcare organisations to identify areas in which they underperform, leading to performance improvement, which will ultimately attract more consumers.

Although the basic conceptual framework is simple, the real-world impact of PPR on consumers’ healthcare decision-making processes remains poorly understood [7]. Past research shows that patients do not often use PPR information in their healthcare decision-making [8] because they do not always perceive differences in the quality ratings of healthcare providers and services [9, 10], they do not trust the data [11] or they do not understand it [12]. Instead, many consumers expect their general practitioners (GPs, i.e. family physician, primary care physician) to recommend a medical specialist or hospital on the basis that the GP knows what is best [13, 14]. In several countries including Australia, the Netherlands and the UK, GPs are gatekeepers to secondary care with patients requiring their referral for access [15].

Australia has dual public and private healthcare sectors. All citizens have free access to a universal public system funded through the taxpayer funded Medicare scheme [16]. Voluntary private insurance reduces access fees to the private healthcare system [17]. The referral process involves a consultation with a GP to discuss medical specialist and hospital options in public or private hospitals. The GP then refers the patient to a medical specialist in an outpatient clinic at a public hospital or to a private specialist clinic at a private office, public or private hospital and the patient is placed on a waiting list for an outpatient consultation with the medical specialist. Following the consultation, the patient will be place on another waitlist for inpatient treatment if required [18]. In theory, GPs can refer a patient to any medical specialist and hospital that the patient chooses to attend. In practice, choice of medical specialist and public hospital are limited in the public system as public hospitals preferred to accept patients within their geographical catchment areas [19]. In the private system, patients can exercise choice in their medical specialist and place of care [17], although some private health insurance organisations have preferred healthcare providers to reduce out-of-pocket expenses [20]. As such, GPs play an important intermediary role in connecting patients with hospitals.

There has been little research examining whether the availability of PPR of hospital data influences GPs’ referral behaviours; the majority of the PPR literature focusses on consumers and healthcare providers such as surgeons and hospitals [8, 21, 22]. Among the few studies conducted in Europe, researchers have found that GPs never or rarely used PPR information when referring patients to hospitals [14, 23–25]. Instead, GPs relied mostly on informal sources of information such as: distance to the hospital; feedback from patients and colleagues; prior experience; and personal contacts with medical specialists, departments or hospitals [14, 25, 26]. Reasons cited by GPs for not using PPR information when selecting a hospital included: lack of awareness of the existence of PPR; lack of motivation to use PPR information; uncertainty regarding how PPR data can be used as a support tool to improve patient outcomes; and concerns about the validity and reliability of the data [14, 24]. While these studies provide useful information on GPs’ referral considerations and use of PPR, they employed quantitative methodologies which do not allow for a deeper understanding of the barriers and facilitators of PPR. They lack insights on how to improve PPR information and its accessibility for GPs, as well as how PPR could better inform GPs’ referrals to hospitals and improve outcomes for patients. Furthermore, they were conducted in Europe, primarily in the Netherlands, so the findings might not apply to Australia given the different healthcare systems. The Netherlands has a single compulsory insurance scheme in which private health insurers and healthcare providers compete with each other for health insurance contracts and patients. Consumers have free choice of health insurer and practitioner [27].

In the PPR literature, GPs have generally been overlooked as potential users of PPR of hospital data. As gatekeepers to secondary care, GPs are responsible for discussing care options with patients and for referring patients to hospitals to receive treatment. GPs’ use of PPR to guide healthcare decision-making has the potential to substantially increase the impact of PPR in meeting its objectives of increased transparency and accountability within the healthcare system, informing healthcare decision-making, and potentially improving the quality of hospital services. Given the lack of current empirical research and generalisability from the few studies conducted in this area, and the increased availability of PPR of hospitals data in Australia which can inform healthcare decision-making, there is an opportunity to review GPs’ current referral processes. The aim of this study was to examine GPs’ awareness and use of PPR of hospital data when referring patients to hospitals, GPs’ perceptions of PPR of hospital data qualities and usefulness, and factors that facilitate or constrain GPs’ use of PPR of hospital data.

## Methods

### Design

This study is part of a larger research program which aims to improve understanding of how PPR might improve quality of care in public and private hospitals in Australia by examining the perspectives of multiple stakeholders. Previous components of the research program included interviews with healthcare consumer advocates, providers, purchasers (public and private funders of healthcare services) [28], and senior hospital clinical administrators. This component of the research program used a qualitative approach to capture the experiences and attitudes of GP participants. We conducted semi-structured interviews with GPs using an interview guide. The interview guide was designed to elicit understanding of: the decision-making underpinning GPs’ patient referral practices and the role of PPR in that process; GPs’ opinions about the accessibility, comprehensibility and utility of PPR information; their perceptions of the MyHospitals website as a prominent example of PPR; and how PPR of hospital data might be improved to better suit GPs’ needs [see Additional file 1]. Participants were invited to make additional comments to ensure that all topics they wished to discuss were covered.

### Recruitment

Recruitment of GPs occurred in Victoria, Australia via the Victorian Primary Care Practice-Based Research Network (VicReN) and GP teaching practices in Victoria. VicReN is a collaboration between the Primary Care Research Unit, Department of General Practice at the University of Melbourne, and primary care practices around Victoria, Australia [29]. VicReN membership is voluntary, its aim is to link members with research projects to build their research capacity. An email describing the study was sent by the VicReN co-ordinator to 131 GPs. A second reminder email was sent a month after the first email. To increase participation rate, an email invitation was sent to 294 GP teaching practices in Victoria. GP teaching practices are practices in which GPs have voluntarily agreed to take medical students from the Department of General Practice at the University of Melbourne for placements. The number of GPs working in each teaching practice was not known. In total, 131 GPs from VicReN and GPs at 294 teaching practices were invited to participate. It is not known how many GPs at each teaching practice viewed the recruitment invitation, nor which recruits were from teaching practices. Each GP received two gold class movie vouchers as compensation for their time participating in the study. Recruitment ceased after four months when 40 participants had been interviewed.

### Data collection

The interviews were undertaken between June and September 2016 by one researcher. Most participants were interviewed via telephone (*n* = 39), but one was conducted face-to-face. Interviews averaged 21 min in length (ranged from 9 to 34 min). All interviews were audio recorded with the GP’s consent. A definition of PPR information was provided at the start of each interview: i.e. “information on the quality of hospitals and/or health-care providers, which is accessible to everyone”.

### Data analysis

Interview recordings were transcribed verbatim and imported into QSR NVivo11 for coding and storage [30]. Thematic analysis was used for identifying, analysing and reporting pattern (themes) within the data [31]. Two researchers independently analysed five interview transcripts. The resulting coding trees (theme lists) were then compared and refined through discussion between the researchers, leading to the development of an agreed coding tree. The remaining interview transcripts were then coded by one researcher, and emergent themes added as needed. For theme development and revision, similar codes were clustered together and subsequently collapsed into emergent themes. The researchers discussed the emergent themes identified from the data until consensus was reached.

### Ethical considerations

Ethical approval for this study was granted by the Melbourne School of Population and Global Health Human Ethics Advisory Group, The University of Melbourne. Written consent was obtained from all GPs prior to data collection to record and use their interview data.

## Results

### Participants

Table 1 shows the demographic characteristics of the 40 GPs interviewed. The gender balance was fairly even (55% female), ages were spread between 30 and 69 years old, 30% were primarily overseas trained, time in practice ranged from 1 month to 43 years, some worked part-time (40%), and most worked predominantly in group practices (95%) located in metropolitan areas (87.5%). The sample were fairly representative of the GP population in Victoria except for gender (female) and location (metropolitan) which were slightly over-represented in this study [32]. The varied ages, training and years of experience of the GPs ensured that opinions were gathered from informants with a variety of experience.

**Table 1:** Demographic characteristic of GPs

	N (%)
Gender	
*Male*	18 (45%)
*Female*	22 (55%)
Age groups (years)	
*30–39*	10 (25%)
*40–49*	7 (17.5%)
*50–59*	16 (40%)
*60–69*	7 (17.5%)
Education & training	
*Australia*	28 (70%)
*Overseas*	12 (30%)
Work status	
*Full-time*	24 (60%)
*Part-time*	16 (40%)
Type of GP practice	
*Group*	38 (95%)
*Solo*	2 (5%)
Location of practice	
*Metropolitan*	35 (87.5%)
*Regional/rural*^*a*^	5 (12.5%)
Years in practice (mean)	20 (range 1 month to 43 years)
Years in practice	
*0–5 years*	8 (20%)
*6–10 years*	4 (10%)
*11–15 years*	2 (5%)
*16–20 years*	2 (5%)
*21+ years*	24 (60%)

^a^Regional and rural locations were combined due to the small number of cases in each location

### Themes

Six main themes related to PPR were identified during analysis: (1) lack of awareness and accessibility; (2) limited utility; (3) lack of trust; (4) unintended consequences; (5) aspirations to strengthen PPR; and (6) healthcare system issues. While the themes are not entirely mutually exclusive, they are elaborated on below under the six theme headings. In doing so, a broader picture emerges of GP decision-making related to their referrals of patients to hospitals.

### Lack of awareness and accessibility

The majority of GPs reported that they were not aware of any specific sources of PPR. In particular, most had not heard of the MyHospitals website. GPs did not have the time to look for PPR during their consultations nor did they have the inclination to search for it: “In my whole experience as a GP in over nearly 25 years I’ve never ever looked at anything like that (PPR).” (GP1). GPs also did not know where to find PPR information and considered access to it difficult:

I wouldn’t know how to access it (PPR information), that’s the honest truth. Even if I was interested, it’s certainly not easily available to me. I don’t see it regularly. You know we get faxed through all sorts of things which we see and we acknowledge but certainly I’ve never come across any flyer or any advertising about how you access those reports. (GP33).

Among the few GPs that were familiar with PPR, they cited the main sources of PPR information as newspapers, the MyHospitals website, and the Department of Health and Human Services’ hospital quality reports.

When I’ve ever seen comparisons across hospitals it’s actually been when it’s been reported in the (news)paper. So if I’m reading the paper and there’s something about that I will take some notes of it – but I don’t seek out that information specifically. I suppose because I didn’t know (it existed). I don’t know why I haven’t (sought it out). I think because maybe I didn’t realise that the information’s there, although of course it is; but also, I suppose, I don’t know if it’s really going to change how I refer. (GP26).

GPs’ lack of awareness of PPR put focus on the information sources that they did use to inform their decision-making around patient referrals, as the following quote highlights:

I wouldn’t particularly sit and look at people’s statistics or hospital statistics. (…) I’d either send them (patients) to a special unit because of the expertise available, or to a surgeon, for instance, or a physician I’d already used and had a good response from – both clinically and patient satisfaction. So I wouldn’t go through statistics and look at, you know, infection rates or death rates for a consultant, orthopaedics or anything like that. (GP20).

When referring patients to public hospitals, GPs generally used their previous experiences (i.e. personal contacts with networks, colleagues, medical specialists and hospitals), their knowledge of patients’ experiences and satisfaction and patients’ geographical catchments area for public hospitals (i.e. distance to the hospitals).

### Limited utility

Many GPs failed to see how PPR information could be utilised in their decision-making processes for public hospital referrals. It was questioned how it would be advantageous and worthwhile to “go wading into a report about public performances of public hospitals?” (GP1). Some were also concerned that such information does not account for patients’ complexities such as social, geographic and economic circumstances (e.g. older patients, patients who value staying close to family, low socio-economic background, low health literacy), so it was considered of limited value to the referral process:

I suppose one of the things is that, like everything, if you look for the hospital that’s the best it may not be the easiest for the patient. It may not actually be better in the whole overall care of the patient, because of either logistics or location or cost. So you have to take more into account than just the hospital. (GP27).

Despite many GPs reporting a lack of awareness and usage of PPR, when the MyHospitals website was described to them, most considered it a valuable tool for accountability, transparency and quality improvement activities in hospitals:

I think it (having PPR) would mean that the public hospitals were more accountable for what they were doing. It would also mean that you (would) know if one hospital was not performing adequately, or was not on par with other hospitals, you could look at why. Is it resources? Is it the patient population that lives around there? – in which case, you know, do we need to have more clinics? So I think overall it’d be beneficial (to have access to PPR data). (GP28).

Some GPs also noted that PPR may be a useful general public resource for informing and managing patients’ expectations of non-urgent clinic appointment and elective surgery waiting times. However, they did not think PPR would inform public hospital choice as public hospitals preferred to accept referrals from patients who live within their geographical catchment area. These GPs did not acknowledge that in certain circumstances, referrals outside a patient geographical catchment area are possible.

I don’t see that it has a great deal of utility for a GP. I think because we’re so location based you’re hamstrung into using the facilities that are closest to you. (…) So even if you want to go for the best and the shiniest clinic, or whatever it is that you think your patient needs, you may be declined entry into that hospital purely based on where your patient lives. (GP26).

### Lack of trust

After it was described to them, some GPs looked at the MyHospitals website during the interview, thus increasing the number of GPs who commented directly about the website. Overall, the majority of GPs questioned the integrity of PPR information – such as that found on the MyHospitals website. They were unclear about how the data were collected, how the quality indicators were calculated, and concerned about data being outdated rather than provided in real-time. They also raised concerns about its reliability and validity including issues of data falsification by hospitals:

If I felt the complication rates were accurately reported, yes I’d take it into account but I’d have to trust how that was reported and I don’t know if hospitals are accurately reporting their complication rates because it’s not something surgeons like to admit to. (…) So I’d have to feel like I trusted that data before I took it on board. (GP9).

The thing about waiting times in emergency, I know how sometimes the consultants are actually making sure the numbers look good where, in fact, they’re actually just moving around patients. I don’t think always good performance means that they have better patient care. I’m cynical about the numbers, because having worked in hospitals, you can work in a place that has very good numbers and things like that, but in fact the care wouldn’t be as good as another place that doesn’t. So I’m quite cynical about their reporting. (GP27).

Publicly reported quality indicators were also deemed to be irrelevant, lacking in meaning, and difficult to interpret as PPR was likely to be “written in language that is hard to understand and very much from a bureaucrat point of view” (GP1). For example, waiting time for elective surgery on the MyHospitals website does not take into account the time from when patients are referred by the GP to the medical specialist; rather, the published waiting time begins only when the patient has been seen by the medical specialist, thus underestimating the total waiting time. Some GPs thought that given they were having difficulties understanding the information, then it was highly likely to cause greater confusion for patients accessing the information:

I’m a little bit concerned about that (MyHospitals) from a scaring-a-patient point of view, or from them not having an understanding of what that means, or how that’s relevant to them on an individual basis. I can see that (MyHospitals) potentially creating some headaches (for patients and GPs). You know like if a patient were to look on the thing and go: “Oh, you know, hospital X has more infections” than whatever, and they might not necessarily understand where those infections occur or how that’s relevant to them when they’re going to a particular clinic. (GP36).

Despite criticisms about the indicators, some GPs said they would consider using PPR information in their future public and private hospital referrals if they thought the indicators to be relevant, specific, real-time, and trustworthy. This was said with the caveat that the use of such information would be highly dependent on whether public hospital referrals outside of patients’ geographical catchment areas and mandatory PPR for private hospitals would be permitted.

### Unintended consequences

GPs expressed concerns that unintended consequences may occur due to PPR. They suggested that PPR could lead to patient selection bias where hospitals or surgeons reject difficult patients whose treatment was determined as risky and could potentially increase (worsen) their complication and mortality rates:

It [PPR] may dissuade the surgeons from taking on difficult cases because difficult cases die more often and the figures will make them look worse; so the only disadvantage of doing that is that some surgeons would just say: "You’re too hard to operate on". The most life threatening cases may end up not being offered surgery. (GP23).

Some GPs perceived that short waiting times in particular hospitals might ultimately increase overall waiting times if GPs favoured referrals to that site. For example, a GP recalled that “a few years ago, Hospital X improved its waiting time for orthopaedics and the word got out and everybody sent their patients to Hospital X, and rather rapidly they got these patients from all over the place coming in” (GP7).

Some GPs were particularly concerned that PPR might also create high demand for services and increase burden in high performing hospitals, whereas low demand in lower performing hospitals might limit their opportunity to improve their processes and patient outcomes. PPR was seen as “enormously helpful (…only) if you were in a situation where there was an oversupply of services (where) people would be keen to show off that they were brilliant” (GP13). As there is not an oversupply of services, there was concern that PPR could push hospitals to manipulate their data to maintain their reputation and prevent an imbalance of patient burden across hospitals: “You obviously can’t show you’re significantly out of kilter with everybody else because that will produce a rush on health services” (GP13); and “If a hospital is performing badly who wants to advertise that? Who wants to publicly notify the general population about it?” (GP29). Creating fear and anxiety through publication of poor performance data among patients who might not have a choice but to use a poor performing hospital (e.g. in regional and rural areas) was of particular concern. Although it was conceded that “it's in everyone’s right to know how any centre is performing per se, let it be hospital, let it be clinic, let it be public or private” (GP29).

### Aspirations to strengthen PPR

Regardless of the types of PPR information currently available, GPs considered waiting times for triage, waiting times for first appointment and surgery, infection rates, complication rates, mortality rates, readmission rates, out-of-pocket costs, patients’ experiences, patients’ satisfaction and complaints to be important indicators that should be publicly reported. The current reporting of wait times for elective surgery were said to be “ridiculous” and in need of improvement (GP2).

The public reporting of patient experience, satisfaction and complaints information was considered particularly useful for consumers, with one GP saying it would be useful information to inform his/her own healthcare decision-making:

I suppose what I’d be interested in, in the case of surgery, is complication rates, personal reports of patients that have received care; similar sort of things to what you do with travel when you’re assessing a hotel. (…) Yes, personal experiences, saying what they have found good about the hospital, what they didn’t find good about the hospital. If I was assessing that hospital for my own personal use, I would find that useful. (GP21).

GPs generally preferred information to be reported at the individual surgeon and clinical unit level instead of hospital level because the latter could mask differences among surgeons and clinical units.

I’d like to see surgeon figures that would be the most important thing for me to see surgeon’s complication rates. Information on complication rates would be really, really good. It can be done in such a way as to not make surgeons feel as if they are being scapegoated. (GP23).

However, they acknowledged that patients are unlikely to know who their surgeon will be in a public hospital, and that PPR information will also need to be case-mix adjusted for appropriate comparisons to be made. Furthermore, GPs urged for better promotion and dissemination of PPR given their lack of awareness of its existence. It was suggested that awareness could be increased via snapshot summaries of PPR data (i.e. faxed, mailed or emailed fliers from hospitals, Primary Health Networks, Royal Australian College of GPs, Australian Health Practitioner Regulation Agency, or government health departments), and a PPR website with a dedicated section targeted to GPs:

I’m just looking at this (MyHospitals website). I work with so many different hospitals and I don’t know if I’d go through and look at every hospital and then compare. I’d probably just want to know what’s the access like at my local hospital compared to the next local hospital for maybe my special interest or specifically for clinics. (…) I don’t really have the time with the patient load to wade through and look through all these different things, I really just want a snapshot. (GP15).

### Healthcare systems issues

A number of GPs discussed PPR issues related to private hospitals and patients’ limited choice of public hospitals, particularly in regional and rural areas. They remarked that PPR information would be more valuable in private hospitals than public hospitals where patients can exercise greater hospital choice:

In the private hospital the consumer has rights, and consumers get choices and the consumer can dictate outcomes, which preferences they have. But public hospital patients don’t get any of those rights. So what’s the point of having a computer program that tells you “look you get better service at hospital X” if hospital X just won’t take you? (GP37).

GPs were especially interested in private hospitals disclosing their specialists’ fees and out-of-pocket costs. GPs also recognised that having accurate waiting times for elective surgery in public hospitals (from GPs referral to specialist to actual surgery) may be helpful in persuading patients to self-fund privately if the waiting time was deemed too long. Some GPs noted that although patients appeared to have greater choice in the private market, limited choice of surgeons and private hospitals could be imposed by private insurance organisations that have preferred healthcare providers.

In addition, GPs working in regional and rural areas recognised the lack of choice in specialists care and public hospitals. GPs noted there were only one or two public hospitals and one private hospital available in regional and rural areas:

The other issue in the country (regional and rural areas) is availability, we only have one cardiologist that we can refer to, we only have one physician who comes to our small village, we only have one surgeon that comes so if they want to be treated in town locally without having to leave the town they’ve only got one surgeon or one physician. And there’s no other choice. (GP23).

## Discussion

Very few of the GPs that contributed to this study had heard of MyHospitals or other sources of PPR, and one had considered using MyHospitals when making patient referrals to public hospitals. These findings are consistent with past studies conducted in Europe which also found that despite the availability of PPR of hospital data, it was little utilised for hospital referrals by GPs [14, 23–25]. Despite the different healthcare systems, the similar findings are largely attributed to the lack of awareness about PPR information among GPs. A contributing factor might be that GPs, internationally, tend to have limited time within or outside of consultations to actively access and make sense of PPR Lack of perceived value in how PPR can inform healthcare decision making and improve the quality of healthcare services, as well as being unclear whether it is the GPs’ responsibility to inform patients about PPR of hospital data may have prevented GPs from using it in their referrals. Our findings, that many of the interviewed GPs did not trust PPR data and did not consider it useful or easily accessible, also accounts for why GPs were not using it when referring patients for secondary care. These findings suggest lack of engagement with GPs in the design and development of PPR systems that GPs would consider trustworthy, useful and accessible. These results also indicate that across our sample of GPs, there has been poor promotion and dissemination of PPR information to this important target user group. Greater PPR resources targeted at GPs may encourage its use and lead to improvements in usability.

Referrals to hospitals, as described by our participants, are largely based on experience, including professional networks, word-of-mouth (what patients reported back to them in follow-up visits) and ease of habit. Similarly, international studies have shown that the most common factors influencing GPs’ referral decisions to healthcare providers or hospitals were their own experience with the medical specialists or hospitals, feedback from patients and professional network colleagues, and distance to the hospital [14, 23–26, 33–36].

GPs’ were reluctant to use PPR data because of concerns about reliability and validity, particularly related to the data being potentially unreliable, outdated and difficult to interpret. This is consistent with past studies which have shown that GPs tend to distrust PPR information as they perceived it to be invalid and unreliable due to the potential that it has been manipulated to achieve hospital targets [14, 24]. In Australia, there has been evidence of manipulation of waiting times in emergency departments and reclassification of patients on elective surgery waiting list [37]. However, it is unclear whether GPs mistrust of PPR data stem from credible evidence or a prejudice against the utility of PPR data given that the majority of GPs were not aware of the MyHospitals website. Future research is warranted to explore the mistrust of PPR data by GPs.

Although GPs did not use PPR data in their referral practice, they were aware of some of the discourse that surrounds the debate of PPR [38, 39]. GPs recognised both the potential benefits of PPR such as promoting accountability, transparency and quality improvement activities in hospitals and the potential unintended consequences of PPR such as overburden of high performing hospitals and increase waiting times which may ultimately impact service delivery, patient selection bias and a decline in public trust by inciting fear among those who may have limited choice of hospitals. These potential adverse outcomes concerns appear valid. Several reviews found some evidence that PPR may be associated with avoidance of high risk patients by doctors [21, 40] and a loss of confidence among the public [41]. Reporting of poor hospital performance may erode public trust at first; however, PPR can highlight deficiencies in healthcare delivery and drive hospital to perform better and therefore restore public confidence. GPs need to consider whether the benefits of using PPR data when referring patients to hospitals outweigh the risk of adverse outcomes. Minimising potential unintended consequences and monitoring these are important to promote trust and increase PPR usability among GPs and the public.

Healthcare systems issues such as public hospital preference for patients living within their local catchment area [42, 43] and limited choice of public hospitals for consumers in regional and rural areas were additional reasons why GPs would not use PPR information. Although these systems issues hinder the use of PPR for informing consumer choice, PPR can increase transparency and accountability, and protect patients by stimulating healthcare quality and performance improvements if hospital staff are aware that GPs and consumers are responsive to PPR information. In support, past studies showed quality improvement occurred among healthcare providers who were concerned that PPR would affect their reputation [44, 45].

Furthermore, GP’s use of PPR can potentially increase the impact of PPR systems by increasing knowledge and trust about good and bad aspects of hospitals, encouraging consumer engagement in their healthcare, and choosing the better performing sites when appropriate or available. However, GPs may not have consider PPR data as part of their responsibility because they have not been educated to consider PPR and its various potential benefits when making referrals – so it remains an untapped source. Past research has shown that GPs are unclear about their role in using PPR data during consultation with patients [24]. Some GPs did not considered their responsibility to advise patients on PPR data, whereas other GPs viewed discussing PPR data with their patients during the referral process as part of their role to support decision making [46]. Future research is warranted to explore whether GPs’ use of PPR data to inform hospital referrals ought to be part of their role. Assessing the comparative priorities of referral information, including hospitals proximity, familiarity and PPR data, using Delphi method or discrete choice analysis will also be helpful to ascertain the level of PPR importance.

### Recommendations

This study, supported by local and international literature, illustrates that when an effective promotion and dissemination strategy for PPR, aimed at GPs, is lacking, that opportunity is lost to increase the impact of PPR. To increase awareness of PPR among GPs, PPR websites and reports should be widely publicised, published in accessible formats, easily understood and made readily available. Although we explored GPs’ perceptions of their PPR needs and desired forms of delivery, it was beyond the scope of the study to comprehensively assess the design and comprehensibility of current PPR websites and reports. Future research is warranted to evaluate the appropriateness of the user interface and contents of PPR for GPs.

Involvement and collaboration with GPs is required to better target and tailor PPR to their needs to increase their accessibility and usability of PPR information. Research suggests that the routine collection and public publication of certain measures, such as patient reported experience and outcome can increase the perceived usability of the data [47, 48]. Such measures are routinely reported in England [49, 50], the Netherlands [51, 52] and the US [53, 54] as a mechanism to incorporate patient perspectives in quality improvement and to promote choice. These measures are found to be positively associated with delivery of care [55], clinical outcomes [56], clinical effectiveness and patient safety [57]. Similarly, the GPs in our study proposed inclusion of such measures which they considered to be more meaningful than those currently available.

Increasing data transparency is essential to improve its perceived trustworthiness among GPs. Educating GPs about quality indicators, PPR methodologies, and ways to discuss PPR with patients should result in more willingness by GPs to use PPR when referring patients to hospitals. Developing real-time data collection, analysis and reporting using smart management systems will facilitate the use of such data to guide decision-making. In Victoria, the establishment of an independent health information agency to analyse and share information across the health system has recently been foreshadowed [58] following a 2016 review of hospital safety and quality assurance in Victoria [59]. The successful establishment of such an agency could enhance the perception of trust if GPs can be convinced that reported data is not being manipulated by hospital staff.

Incorporating PPR data from the public domain into electronic referral systems may also increase the use of PPR data by GPs in their referral processes. For example, in England and the Netherlands where GPs are also gatekeepers to secondary care, systems such as ‘Choose and Book’ and ‘ZorgDomein’ have been implemented to support patient choice at the point of referral [60, 61]. Both electronic referral systems include a directory of medical specialists, clinics in the hospitals and average waiting times. Technical challenges and GP resistance to the system, at least in the English experience, were identified during the implementation which limited its execution. Additional investment in IT infrastructure and remuneration for GPs by the government were required to encourage GP uptake of the system [62]. Overall, electronic referral systems have the potential to improve GPs’ referral decisions by including PPR data and involving patients in the process. Mandating PPR of private hospital data in the same way as public hospital data provides necessary information to enable choice in areas where choice can be exercised [59]. Currently, only 35% of Australian private hospitals participate in PPR on the MyHospitals website, and they do not necessarily report on all of the indicators required by public providers [4]. Some private healthcare providers publish their own PPR websites to help consumers make informed decisions [e.g. [63]]. Although a positive step towards PPR, it does not assist GPs and others who want to be able to locate such information in one central place.

### Limitations

The limitations related to this study should be considered when interpreting the findings. Recruitment, based on GPs members of VicReN and GP teaching practices, may have resulted in a biased sample. Given the small numbers and under-representation of GPs working in regional and rural areas, the findings are not generalisable. However, the sample of GPs, recruited via VicReN and GP teaching practices, consist primarily of GPs who are interested in research, therefore, they might be more likely to be actively engaged in learning and new knowledge, such as PPR makes available, than non-participating GPs. Given that many GPs were not aware of PPR data; their perceptions of PPR utility and data quality are likely to be limited. MyHospitals was given as the prominent example of a source of PPR and GPs perceptions of PPR may have been skewed towards that, with little comment on other sources of PPR information. Finally, a conclusion cannot be reached on what the GPs thought of PPR overall given that many GPs were unaware of specific example of it. Some GPs viewed and commented on the MyHospitals website during their interviews; their opinions were based on first impressions rather than a working knowledge of the website.

## Conclusions

Given that GPs are important intermediaries between healthcare consumers and hospital care, it is of great importance that they are well informed when guiding patients in their treatment decisions and referrals to target the better performing services where possible, or to alert patients to potential quality or safety problems as indicated by PPR data. Such openness and transparency could potentially trigger quality improvement at poor performing hospitals and build patient trust and engagement with healthcare. An apparent obstacle to overcome if GPs are to better utilise PPR information is ensuring that systems of PPR are developed with collaboration from GPs to ensure that the reported performance quality indicators, formats and mediums serve their information needs. Developing systems of PPR tailored to meet the needs of various target audiences (such as GPs, consumers, hospital staff and so on) should be considered to increase the usability and impact of PPR.

## Additional file

Additional file 1: Interview guide. The interview guide used to elicit understanding of GPs’ referral behaviours and the use of PPR of hospital data. (DOCX 16 kb)

## Abbreviations

AIHW: Australian Institute of Health and Welfare
GP: General practitioner
PPR: Public performance reporting
UK: United Kingdom
US: United States
VicReN: Victorian Primary Care Practice-Based Research Network
