# Supplementary material for: The use of public performance reporting by general practitioners: a study of perceptions and referral behaviours
Source: BMC Fam Pract. 2018 Feb 12;19:29. doi: 10.1186/s12875-018-0719-4 (PMC5810107; doi:10.1186/s12875-018-0719-4)
Supplement: Supplementary file 1 — Interview guide. The interview guide used to elicit understanding of GPs’ referral behaviours and the use of PPR of hospital data. (DOCX 16 kb) [file 12875_2018_719_MOESM1_ESM.docx]

**Interview guide**

We are looking to better understand the role of GPs in the decision making process of referrals and whether the decision they make is influenced by the availability of hospital performance reporting information. For the purpose of this study, we defined publicly available performance reporting as ‘information on the quality of hospitals and/or health-care providers, which is accessible to everyone’. We are particularly focussed on information presented on the internet that enables comparisons between hospitals.

**Referral considerations**

1. What information do you consider when referring patients who will need hospitalisation (urgent/non-urgent)?
2. Where do you obtain this information from?
3. What do you think are the strengths of your current patients’ referral to hospital?
4. What will help you improve your current patients’ referral to hospital?

**The role of publicly available performance reporting**

1. Are you aware of any specific sources of publicly available hospital performance reporting?
   1. If yes,
      1. In the past 12 months, did you access publicly available hospital performance reporting when referring patients?
         1. If no, why not?
         2. If yes,
            1. What publicly available performance reporting information did you access?
            2. How often did you access publicly available hospital performance reporting?
            3. How easily accessible and comprehensible was the publicly available performance reporting data?
            4. What do you think about the current quality indicators that are reported on?
      2. Have you altered your referrals patterns based on the publicly available performance reporting information?
         1. If no, why not?
         2. If yes, why?
   2. If no,
      1. Have you heard of ‘MyHospitals’ website? [If no: MyHospitals is a government-owned website. The information on this site is for members of the public, clinicians including doctors and nurses. The site allows you to view the performance of public and private hospitals against indicators such as waiting times in emergency departments or for some types of surgery, rates of bloodstream infections acquired in hospital, the length of time patients spend in hospital after being admitted for various conditions or procedures, and other indicators]
      2. What are your thoughts on the role of public reporting of hospital performance data?
      3. Can you think of any advantages for GPs to use public reporting of hospital performance data when they refer patients to hospital?
      4. Can you think of any disadvantages for GPs to use public reporting of hospital performance data when they refer patients to hospital?
2. Have you ever suggested to a patient that they look at public reporting websites like MyHospitals to help inform their decision?
   1. No
   2. If yes,
      1. How often?
3. How could publicly available hospital performance reporting be improved to better suit your patients’ needs?
4. How could publicly available hospital performance reporting be improved to better suit your needs?
5. If someone from your family needed to go to hospital for elective treatment, what information would you like to see publicly reported to inform their choice?
6. Would you be using public reporting of hospital performance data in the future for your patients’ referral to hospital?
   1. If yes, why?
      1. How would you like to receive the hospital performance information?
   2. If no, why not?
7. Is there anything else you would like to add about public reporting of hospital performance data that we have not covered?

**Demographic characteristics**

1. What is your age?
2. What year did you graduate? How long have you been a GP?
3. Where did you do your training? (i.e. Australia or overseas)
4. Do you work full-time or part-time?
5. Do you work in a solo or group general practice?
6. Do you work in a metropolitan or rural general practice?
7. What is the postcode of your practice?
